# Retention of the Catamenia from Obstruction

**Published:** 1866-06

**Authors:** F. C. Robinson

**Affiliations:** Wyanet, Ill.


*Retention of the Catamenia from Obstruction.* By F. C. Robinson, M. D., of Wyanet, Ill.

In February last I was consulted by Miss W., aged 15, upon the non-appearance of the menses, which the lady attributed to cold taken during a former flow. Upon inquiry I learned that she menstruated for the first time in August, 1865, and continued regular until November, when it ceased. Since that time she had had the menstrual molimen every four weeks with much pain and constitutional suffering, increasing in intensity at every successive period, but without any discharge. The girl being so positive that cold was the cause of the suppression, I ordered a cathartic, followed by balsam copaiba, in doses of twenty-five drops every eight hours, hot pediluvial and hip baths, and directed her to inform me if she obtained no relief.

In the evening of March 3d, I was again called to see the lady, who had been getting worse since my first visit, and found her suffering the most excruciating pain in the uterus, abdomen and thighs—at times so severe as to make her rave as in a state of delirium. I mistrusted the cause of her pain to be from obstruction, and stating my fears to the patient, suggested an examination. The external organs of generation were normal; the vagina small and contracted, and an inch and a half from its orifice was completely closed by a firm, elastic membrane, forming a cul de sac, point upwards. I administered chloroform, and using a director and my index finger, ruptured the membrane, causing slight hemorrhage, but, as yet, no relief. Above this membrane the vagina was crossed by several fibrous bands, which I also divided, revealing a distended uterus. It was lower in the pelvis, and pressing upon the neck of the bladder, caused irritation and a desire for frequent micturition, which I learned subsequently. The os uteri was also closed by a membrane similar to the one above mentioned, and which I succeeded in penetrating, using a female catheter, when about sixteen ounces of reddish-brown, tenacious fluid, or menses in a condensed form, escaped. The pain ceased in a few hours, and, except a slight nausea from the chloroform, the girl did well, and was soon able to resume her duties, well satisfied with her condition and the relief she had obtained.

From such information as I can get, it is impossible to diagnose the cause of this double occlusion. The fact of previous menstruation precludes the idea of its being congenital, and there being no evidence of injury, I can only account for it by saying it is an anomaly of nature, governed by the same physiological laws necessary to the building and repair of the human economy, being varied only by the organization of plastic lymph upon a slightly-inflamed mucous surface.

